# TNF‐α induced extracellular release of keratinocyte high‐mobility group box 1 in Stevens‐Johnson syndrome/toxic epidermal necrolysis: Biomarker and putative mechanism of pathogenesis

**DOI:** 10.1111/1346-8138.16847

**Published:** 2023-06-02

**Authors:** Gospel Nwikue, Anna Olsson‐Brown, Nourah Aboheimed, Vincent Yip, Carol Jolly, Andreea Luchian, Lorenzo Ressel, Anurag Sharma, Wilma Bergfeld, Shaheda Ahmed, Anne Dickinson, Munir Pirmohamed, Daniel F. Carr

**Affiliations:** ^1^ Department Pharmacology and Therapeutics, Institute of Systems, Molecular and Integrative Biology University of Liverpool Liverpool UK; ^2^ Department of Veterinary Pathology and Public Health, Institute of Veterinary Science University of Liverpool Liverpool UK; ^3^ Department of Dermatology and Dermatopathology Cleveland Clinic Foundation Cleveland Ohio USA; ^4^ Alcyomics Ltd Newcastle UK

**Keywords:** high‐mobility group box 1, Stevens–Johnson syndrome, toxic epidermal necrolysis, tumor necrosis factor alpha

## Abstract

Decreased epidermal high‐mobility group box 1 (HMGB1) expression is an early marker of epidermal injury in Stevens–Johnson syndrome/toxic epidermal necrolysis (SJS/TEN). Etanercept, an anti‐tumor necrosis factor therapeutic, is effective in the treatment of SJS/TEN. The objective was to characterize antitumor necrosis factor‐alpha (TNF‐α)‐mediated HMGB1 keratinocyte/epidermal release and etanercept modulation. HMGB1 release from TNF‐α treated (± etanercept), or doxycycline‐inducible RIPK3 or Bak‐expressing human keratinocyte cells (HaCaTs) was determined by western blot/ELISA. Healthy skin explants were treated with TNF‐α or serum (1:10 dilution) from immune checkpoint inhibitor‐tolerant, lichenoid dermatitis or SJS/TEN patients ± etanercept. Histological and immunohistochemical analysis of HMGB1 was undertaken. TNF‐α induced HMGB1 release in vitro via both necroptosis and apoptosis. Exposure of skin explants to TNF‐α or SJS/TEN serum resulted in significant epidermal toxicity/detachment with substantial HMGB1 release which was attenuated by etanercept. Whole‐slide image analysis of biopsies demonstrated significantly lower epidermal HMGB1 in pre‐blistered SJS/TEN versus control (*P* < 0.05). Keratinocyte HMGB1 release, predominantly caused by necroptosis, can be attenuated by etanercept. Although TNF‐α is a key mediator of epidermal HMGB1 release, other cytokines/cytotoxic proteins also contribute. Skin explant models represent a potential model of SJS/TEN that could be utilized for further mechanistic studies and targeted therapy screening.

## 1 INTRODUCTION

Stevens–Johnson syndrome/toxic epidermal necrolysis (SJS/TEN) is a rare, immune‐mediated, cutaneous blistering condition, most often caused by drugs and characterized by widespread keratinocyte death and epidermal detachment. Although much is known about the immunopathogenesis of SJS/TEN, gaps in our knowledge of how cell death and epidermal detachment occur still exist.

Previous work^1^ demonstrated that epidermal expression of high‐mobility group box 1 (HMGB1) protein is able to discriminate between severe cutaneous adverse drug reactions (ADRs) (SJS/TEN) and maculopapular exanthema. HMGB1 is a damage associated molecular pattern (DAMP) protein which, in its unacetylated form, acts as a marker of both sterile toxicity and, in its acetylated form, as a marker of innate immune response.^2^

Etanercept is an antitumor necrosis factor‐alpha (TNF‐α) monoclonal antibody used for treating auto‐inflammatory conditions including psoriasis and rheumatoid arthritis. A number of case studies have also suggested that it is an effective treatment for SJS/TEN^3^ and reduces mortality versus current standard of care (high‐dose prednisolone).^4^ Despite reports of rapid re‐epithelialisation in response to etanercept,^5^ there is currently limited understanding of the specific mechanism of action of etanercept in SJS/TEN treatment. Studies suggest that T‐cell derived TNF‐α contributes to keratinocyte cell death via Fas ligand.^6^ However, necroptosis is the key cell death mechanism responsible for the significant keratinocyte death seen in SJS/TEN^7^, ^8^ but its modulation by TNF‐α is yet to be established. Furthermore, the role of TNF‐α in mediating keratinocyte HMGB1 release and the effect of etanercept is not understood.

The aim of this study was to determine if TNF‐α‐mediated keratinocyte cell death is associated with HMGB1 release, both in vitro and ex vivo, and whether this could be ameliorated by etanercept treatment. Furthermore, digital pathology methodologies have been utilized to quantitatively assess previously reported decreased SJS/TEN epidermal HMGB1 expression.^1^

## 2 METHODS

### 2.1 Patient cohort

#### 2.1.1 Immune checkpoint inhibitor ADR patients and tolerant controls

Metastatic melanoma patients experiencing cutaneous ADRs secondary to immune checkpoint inhibitors (and tolerant controls) were prospectively recruited at the Clatterbridge Cancer Centre, Wirral, UK (December 2018 to November 2019).

Blood serum samples were taken at time of reaction (cases) and a comparator sample taken from ICI‐tolerant controls after four cycles of immune checkpoint inhibitor (ICI) treatment. Ethics approval for the hypersensitivity study was granted in July 2012 by the National Research Ethics Service: Committee North West‐ Manchester North (Ref: 12/NW/0525). All patients gave full, informed written consent to participate. Three patients were identified (1 × SJS/TEN,^9^ 1 × lichenoid dermatitis, and 1 × tolerant control; Supporting Information Table S2) whose sera were utilized for exposure to healthy skin explants. Serum TNF‐α concentrations were determined by enzyme‐linked immunosorbent assay (ELISA; R&D Systems) according to the manufacturer's protocol.

#### 2.1.2 Cutaneous ADR skin biopsies

Formalin‐fixed paraffin‐embedded skin samples were identified from the histology archive database at Cleveland Clinic, OH, USA, from 2013 to 2020 using criteria previously described.^1^ Briefly, an internal diagnosis or description search included the terms “Stevens‐Johnson Syndrome” or “toxic epidermal necrolysis” and drug eruption/drug reaction (including “dermal hypersensitivity reaction”). Cases were selected where a diagnosis of drug‐induced SJS/TEN or maculopapular exanthema was very strongly favored and was supported by clinical notes. Normal skin from excision specimens was utilized as healthy control skin. Suspected causal drugs were identified from clinical notes (Supporting Information Table S2).

### 2.2 Creation of HaCaTs stably expressing doxycycline‐inducible cell death regulatory genes

DNA sequences for full‐length human for receptor interacting protein kinase 3 (RIPK3) (NM_006871.3), mixed lineage kinase domain like pseudokinase (MLKL) (NM_152649.3) and Bak (NM_001188.3) were PCR amplified with Phusion High Fidelity DNA polymerase (ThermoFisher Inc.) with both forward and reverse primers containing Sfil restriction sites (Supporting Information Table S1). Sfil restricted fragments were ligated into pSB‐Tet Blast, which contains a tetracycline inducible expression cassette flanked by Sleeping Beauty transposon inverted repeat/direct repeats (IR/DRs) containing ampicillin resistance and blasticidin selection markers.

Human keratinocyte cells (HaCaTs) were seeded overnight in six‐well plates at 2.5 × 10^5^ cells/well in 2 mL of Dulbecco's Modified Eagles Media (DMEM, +10% fetal bovine serum (FBS); both Sigma‐Aldrich). Three micrograms of pSB‐Tet Blast plasmid DNA and 300 ng of pCMV hpyPBx100 (containing the hyperactive Sleeping Beauty Transposase) per well were co‐transfected using JetPEI (101‐10; Polyplus) according to the manufacturer's protocol. Transfected HaCaT cells were treated with 1.5 μg/mL blasticidin (InvivoGen) for 2 days followed by 5 μg/mL (6 days), 10 μg/mL (2 days), and 15 μg/mL (2 days) with fresh drug applied every 48 h. The remaining antibiotic‐resistant population was passaged on as polyclonal HaCaT cells stably expressing tetracycline‐inducible RIPK3, MLKL, or Bak.

### 2.3 HaCaT cell viability

Immortalized human keratinocytes (HaCaT) cells were periodically tested for mycoplasma contamination using an in‐house polymerase chain reaction (PCR) methodology. HaCaTs were seeded into 96‐well plates at 2.5 × 10^4^ cells/well overnight in serum‐free (SF) DMEM (+1% penicillin/streptomycin, 0.01% dimethylsulfoxide (DMSO)). Cells (in serum‐free media) were exposed to 10 ng/mL TNF‐α ± 5 uM BV‐6, ±40 μM necrostain‐1 (NEC‐1), ±50 μM carbobenzoxy‐valyl‐alanyl‐aspartyl‐[O‐methyl]‐fluoromethylketone (Z‐VAD‐FMK) for 24 h or 0.5, and 1 μg/mL doxycycline for 6 h. Twenty microliters of 3‐4,5‐dimethylthiazol‐2‐yl‐2,5‐diphenyltetrazolium bromide (MTT; 2 μg/μL final concentration) per well was added and incubated for 2 h at 37°C. One hundred microliters of lysis buffer 20% sodium dodecyl sulfate (SDS) (w/v) in 50% (w/v) dimethylformamide was added and incubated at room temperature overnight. Optical density at 595 nm was determined by multimode plate spectrophotometry (Beckman Coulter). Cell viability was expressed as a percentage normalized to untreated controls. Details of the flow cytometric analysis of cell death can be found in the Supporting Information.

### 2.4 Flow cytometry

Cells were pelleted (×2) at 2000 rpm for 5 min at room temperature, washed in phosphate‐buffered saline (PBS) and re‐suspended in 1X annexin binding buffer (10 mM 4‐(2‐hydroxyethyl)‐1‐piperazineethanesulfonic acid (HEPES), 140 mM NaCl, and 2.5 mM CaCl_2_, pH 7.5) plus 2.5 μg/mL Annexin V (AV)‐fluorescein isothiocyanate (FITC) and 0.25 μg/mL propidium iodide (PI). Cells were incubated on ice for 15 min in the dark then analyzed using an Attune Acoustic Focusing Cytometer (Applied Biosystems). Polygon gating was performed on 10 000 cells. The BL3 channel was used to capture PI positive cells and the BL1 channel to capture AV positive cells.

### 2.5 Western blotting

Twenty‐five microliters of cell‐free supernatant or 25 mg of total protein cell lysate was resolved on a 4–12% NUPAGE™ pre‐cast SDS‐polyacrylamide gel electrophoresis (PAGE) gel (Life Technologies Inc.) at 90 V (15 min) then 180 V (60 min). Transfer of immobilized proteins to nitrocellulose membrane was carried out at 100 V for 1 h and the membrane was blocked in Tris‐buffered saline‐tween containing 10% dried‐milk powder for 1 h at room temperature. Membranes were probed with rabbit anti‐human high‐mobility group box 1 (HMGB1) primary antibody (1:5000 dilution, overnight; Abcam Ltd) and goat anti‐rabbit immunoglobulin G (IgG) horseradish peroxidase (HRP)‐conjugate secondary antibody (1:10 000 dilution, 2 h; Sigma‐Aldrich).

Streptavidin‐tagged RIPK3 and Bak were probed with strepMAB‐classic mouse anti‐human antibody (1:4000; IBA Life Sciences) and horse anti‐mouse HRP‐conjugate (1:10 000 dilution; Cell Signaling Technologies). Details of all other primary and secondary antibodies used are given in Supporting Information Table S3. Membranes were developed and visualized using enhanced chemiluminescent substrate (ECL) and a Chemidoc Touch Imaging System (both Bio‐Rad). Image densitometric analysis was undertaken using Image J software.

### 2.6 Skin explants

As previously described and published,^10^ 4‐mm biopsies were taken from healthy skin tissue (Tissue Solutions Ltd). Anakinra was utilized as a positive control as previous observations suggest that at 10 μg/mL it induces a toxicity and epidermal separation phenotype in explants akin to SJS/TEN. This is likely comparable to immediate injection site reactions where high local anakinra concentrations induce mast cell degranulation^11^ and can lead to TNF‐induced neutrophil infiltration^12^ and tissue injury. Explants were cultured for 72 h in 200 μL of media (X‐VivoTM 10, Lonza) ±1 μg/mL etanercept (Benepali, Biogen Inc.) containing (i) single patient sera (SJS/TEN, lichenoid dermatitis, or control) diluted 1:10, (ii) 10 ng/mL TNF‐α (R&D systems), or (iii) 10 μg/mL anakinra (*n* = 3 for all groups; Sigma‐Aldrich). A single replicate was snap‐frozen and stored at −80°C in optimal cutting temperature media with the other two formalin‐fixed and paraffin‐embedded for H&E staining and immunohistochemistry. Histological damage was graded according to the Lerner criteria.^13^ Briefly, these are:

- Grade 0: no observable damage to skin keratinocytes
- Grade I: mild vacuolization of basal cells
- Grade II: vacuolization of basal cells and dyskeratotic bodies
- Grade III: subepidermal cleft formation at the dermal‐epidermal junction
- Grade IV: complete epidermal separation.

### 2.7 Serum/supernatant HMGB1 measurement

HaCaT cells were seeded in 12‐well plates (1 × 10^6^/well in 500 uL DMEM +10% FBS +1% pen/strep) for 24 h. Supernatant was spun at 5000 rpm for 5 min to remove cell debris.

Supernatant total HMGB1 protein concentrations were determined by ELISA according to the manufacturer's protocol (IBL International GmBH). Samples failing to reach the manufacturer's lower limit of quantification (0.2 ng/mL) or where replicates were discordant by >15% were excluded. For explants treated with human sera, input HMGB1 (how much was in the original serum sample) was determined and subtracted from the final concentration. Detailed protocols for western blot analysis are available in the Supporting Information.

### 2.8 Immunohistochemistry

Sections (5 μm) were dewaxed and subjected to antigen retrieval in Dako PT buffer high pH (Agilent Technologies Ltd) using a computer‐controlled antigen retrieval workstation (PT Link; Agilent Technologies Ltd) for 20 min at 98°C. Sections were stained for 1 h at room temperature (RT) using an automated immunostainer (Link 48; Agilent Technologies Ltd), with (i) rabbit polyclonal primary anti‐human antibody for HMGB1 (Abcam Ltd; 1:1000 dilution); (ii) cleaved‐caspase 3 (1:50 dilution; Cell Signaling Technology) or (iii) RIPK3 (1:550 dilution; Abcan Ltd). This was followed by a 30‐min incubation (RT) with the polymer peroxidase‐based detection system (Anti Mouse/Rabbit Envision Flex+, Agilent Technologies Ltd). Visualization was with diaminobenzidine (Agilent Technologies Ltd). Consecutive sections incubated with nonimmune rabbit serum served as negative controls. Positive reaction was represented by a distinct brown nuclear (or rarely cytoplasmic) reaction. Positive control was represented by epidermis and follicle in normal skin.

### 2.9 Analysis of whole‐slide images

HMGB1‐stained slides were scanned using a Leica Aperio CS2 scanner at 40× magnification. Whole‐slide image analysis was undertaken using QuPath software.^14^ Epidermis was manually separated from dermis, annotating the area of interest, and appropriate classification attributed to each annotation. Positive cells were identified as those with an evident brown precipitate due to a 3,3'‐Diaminobenzidine (DAB) reaction. Positive cell detection parameters were optimized and analysis run for all classified annotations. Three different thresholds were set to categorize the cells according to staining intensity as follows: negative (blue), weakly positive (yellow), moderately positive (orange), and strongly positive (red) (Figure 4a). Total positive cells were the sum of weakly, moderately, and strongly positive cells. Data presented are HMGB1 positive stained cells for epidermis or dermis.

### 2.10 Statistical analysis

For serum and supernatant HMGB1 concentrations, a one‐way analysis of variance with Bonferroni correction was undertaken. For western blot densitometry, a Mann–Whitney *U* test was utilized. All analyses were performed using Prism 5 software (GraphPad Inc.).

## 3 RESULTS

### 3.1 TNF‐α induced HMGB1 release in vitro

**Figure 1:**
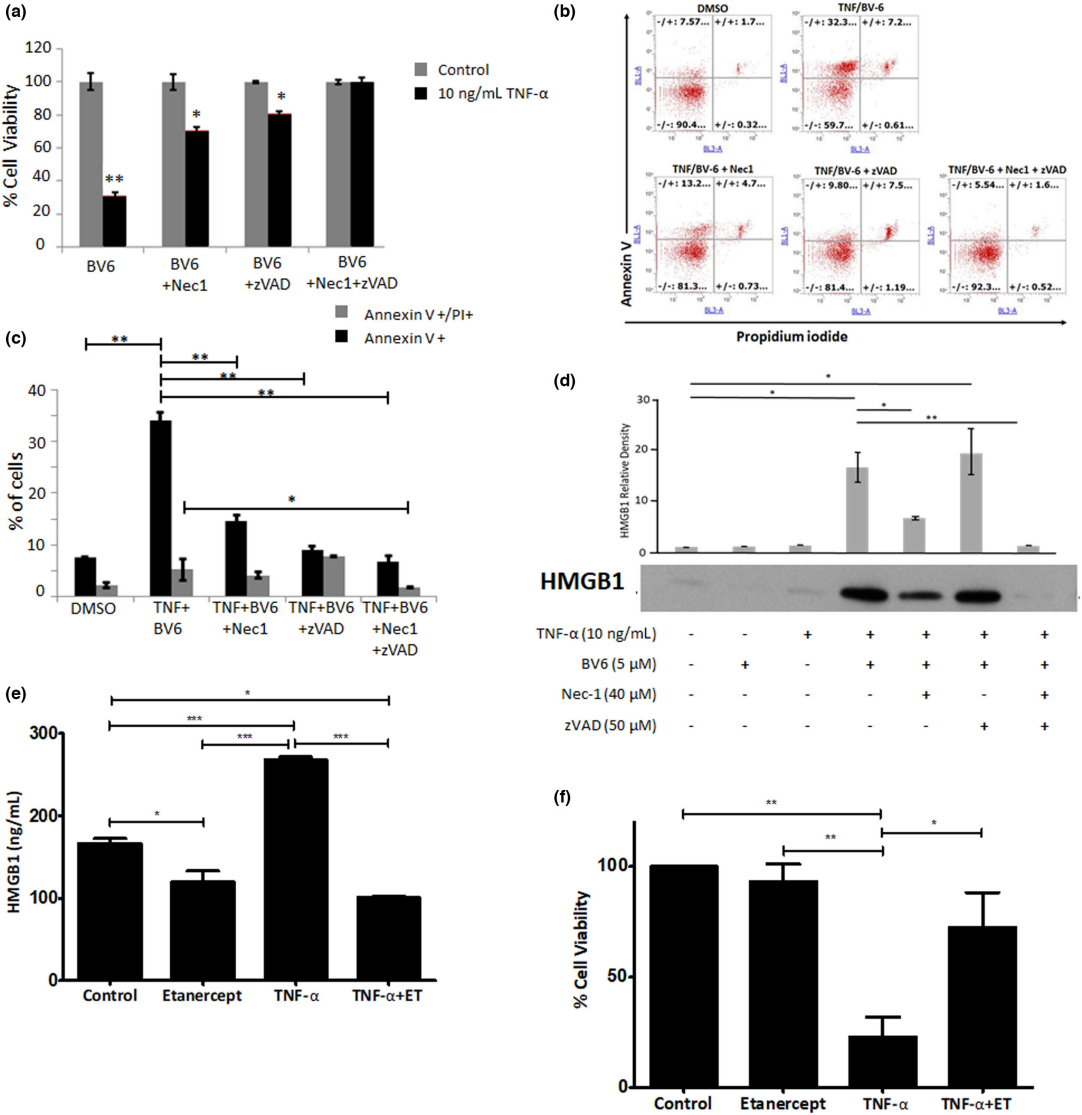
A combination of TNF‐α and BV‐6‐induced HMGB1 release in HaCaTs which is modulated by inhibitors of both necroptosis (necrostatin) and apoptosis (Z‐VAD‐FMK). (a) HaCaT cell viability determined by MTT assay, (b) representative flow cytometric scatter plot of AV‐ and PI‐stained cells with (c) percentage cells positive stained and (d) extracellular HMGB1 following 24 h TNF‐α exposure ±40 μM necrostatin, 50 μM ZVAD (exemplar western blot image selected from *n* = 3). Densitometric analysis was performed on three separate blots and statistical analysis was undertaken using the Mann–Whitney *U* test (*P ˂ 0.05 and **P ˂ 0.01). (e) Supernatant HMGB1 levels determined by ELISA and (f) corresponding HaCaT viability by MTT assay (with BV‐6 pre‐treatment). Data represent mean normalized to untreated control (±SE) of three separate experiments conducted in triplicate (*P ˂ 0.05, **P ˂ 0.01, ***P < 0.001 and # < 0.05 vs. TNF‐a/BV‐6 treated cells).

Our initial work aimed to characterize TNF‐α induced cell‐death and HMGB1 release in a keratinocyte cell line (HaCaT) and determine whether necroptic or apoptotic pathways contributed significantly to HMGB1 release. Since TNF‐α alone did not lead to significant cell death in this model, we pre‐incubated cells with 5 μM BV‐6, a bivalent second mitochondria‐derived activator of caspases (SMAC_ mimetic which promotes auto‐ubiquitination of cellular inhibitor of apoptosis (cIAP)‐1 and ‐2 and X‐linked inhibitor of apoptosis (XIAP) and subsequent degradation by the proteasome. It is thought that this blocks nuclear factor kappa B (NF‐kB) activation at the level of the death inducing signaling complex (DISC),^15^ sensitizing cells to TNF‐α‐induced cell death (Figure 1). This led to a significant decrease in the viability of TNF‐α treated cells versus controls (*P* < 0.01 Figure 1a). The receptor‐interacting serine/threonine‐protein kinase 1 (RIPK1) inhibitor NEC1 (inhibitor of necroptosis) or pan‐caspase inhibitor zVAD‐FMK (inhibitor of apoptosis) partially rescued viability (*P* < 0.05 vs. control). NEC1 and zVAD in combination negated TNF‐α‐induced cell death (*P* > 0.05).

The addition of NEC‐1, zVAD or both decreased the number of AV+ (early apoptotic/necrotic cells) versus no inhibitor (*P* < 0.01; Figure 1b,c). Moreover, zVAD+NEC‐1 treated cells exhibited a significant decrease in PI+/AV+ cells (late apoptosis, *P* < 0.05; Figure 1c). Inhibition of RIPK1 (necroptosis) by necrostatin resulted in a significant decrease in TNF‐α induced extracellular HMGB1 (*P* < 0.05) which was greater than that for zVAD, with use of both resulting in attenuated HMGB1 release (*P* < 0.01; Figure 1d).

**Figure 2:**
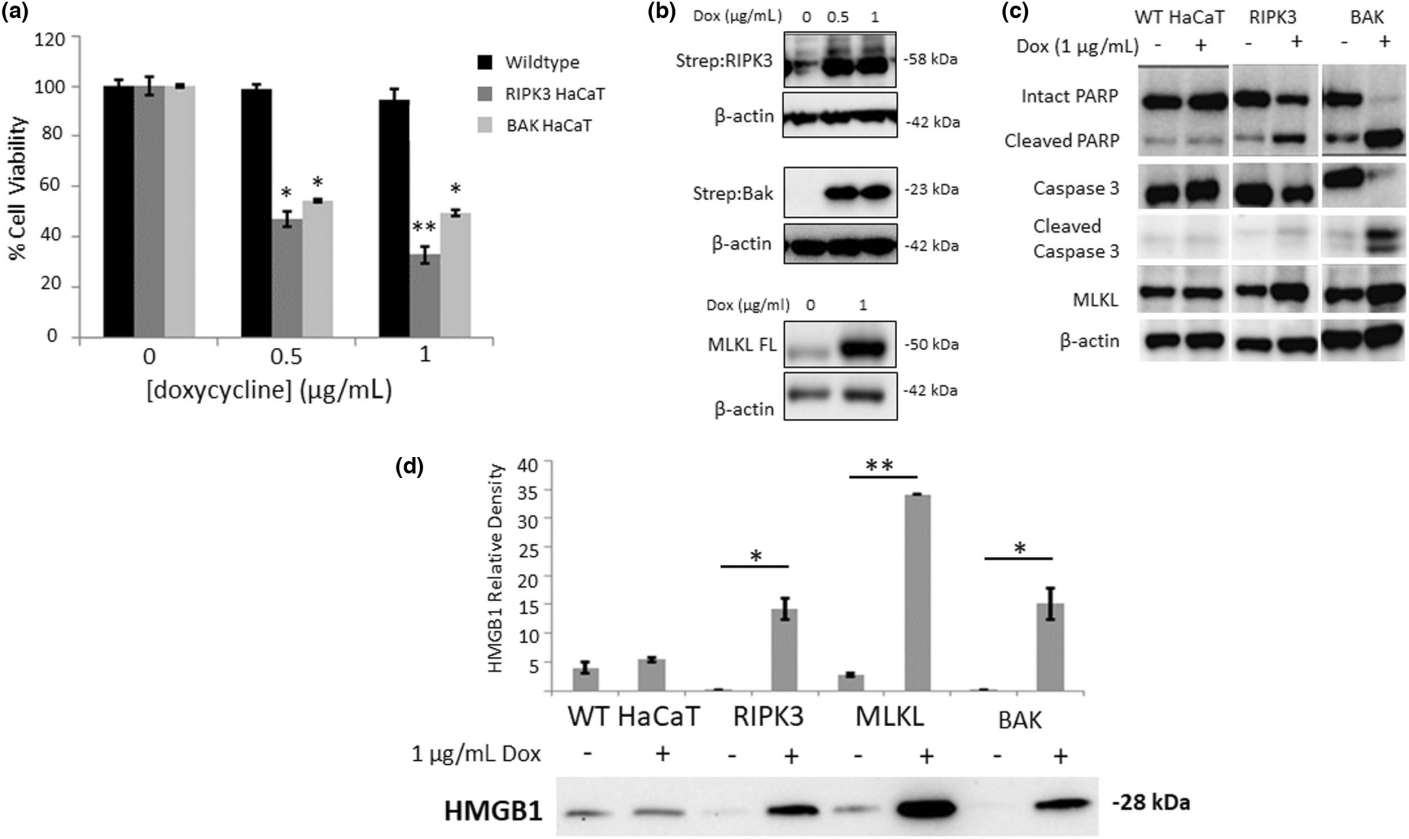
Effect of doxycycline‐induced RIPK3 and Bak induction on (a) HaCaT cell viability (data represent mean normalized to untreated control ±SE, *n* = 3, *P ˂ 0.05, **P ˂ 0.01), (b) corresponding induction of RIPK3, Bak, and full‐length MLKL expression by doxycycline, (c) effect of RIPK3 and Bak expression on necroptotic and apoptotic markers, and (d) modulation of HMGB1 extracellular release following induction of RIPK3, MLKL, and Bak. HaCaT cells were treated for 6 h with 1 μg/mL doxycycline. Exemplar western blot images selected from *n* = 3 (b–d). Images have been rearranged to aid clarity. Densitometric analysis was performed on three separate blots and statistical analysis was undertaken using the Mann–Whitney *U* test (*P ˂ 0.05 and **P ˂ 0.01).

Having established the contribution of necroptosis apoptosis to HaCaT HMGB1 release in a TNF‐α exposed model, we next wanted to observe it in a “cleaner” model of both cell death mechanisms. This utilized HaCaTs stably transfected with inducible necroptotic and apoptotic cell‐death mediators. HMGB1 release was assessed in HaCaTs stably transfected with inducible necroptotic (RIPK3 and MLKL) and (intrinsic pathway) apoptotic (Bak)^16^ cell‐death mediators. Doxycycline‐induced expression of both RIPK3 and Bak induced a statistically significant decrease in cell viability (Figure 2a) commensurate with increased expression of the respective genes (Figure 2b). Increases in PARP and caspase 3 cleavage were observed in Bak overexpressing HaCaTs but not those overexpressing RIPK3. In RIPK3 overexpressing cells increased MLKL expression was additionally seen (Figure 2c). Extracellular HMGB1 expression was significantly induced in all three dox‐inducible cell‐lines (compared to wild‐type, *P* < 0.01), with MLKL expression appearing to exhibit the highest levels (Figure 2d).

### 3.2 TNF‐α induced HMGB1 release and modulation by etanercept

Having establish a predominant role for necroptosis, in both our TNF‐α exposed and cell‐death inducible models, we next wanted to quantify HMGB1 release and assess the effect on it of etanercept (a TNF‐inhibitor used in the treatment of SJS/TEN^4^) in our in vitro model. TNF‐α induced a significant increase in HMGB1 extracellular release from HaCaT cells (mean [±SE], 268.2/mL ± 3.2 vs. 166.8/mL ± 5.8 [control] and 120.1 ng/mL ± 12.8 [etanercept only], both *P* < 0.001) (Figure 1e). This was negated by the co‐administration of etanercept with TNF‐α (100.8 ng/mL ± 2.0, *P* < 0.001; Figure 1e). Conversely, TNF‐α decreased cell viability (23.0% ± 8.9) compared to normalized control incubations (*P* < 0.01), and this was significantly rescued by etanercept (72.5% ± 15.7, *P* < 0.05) (Figure 1f).

**Figure 3:**
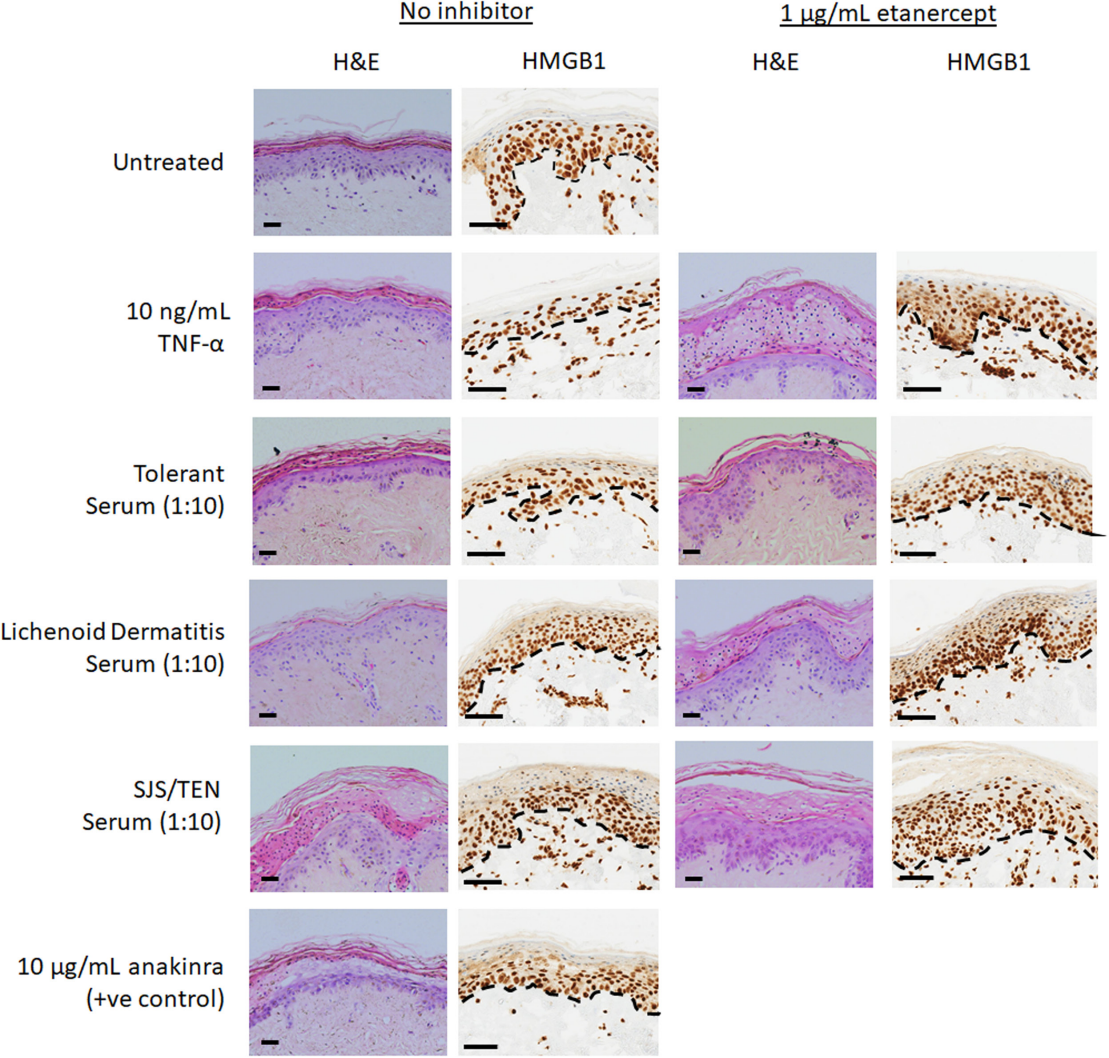
Effect of 72 h of 10 ng/mL TNF‐α or cutaneous ADR patient serum (control, lichenoid dermatitis or SJS/TEN) exposure ±1 μg/mL etanercept on healthy skin explant morphology (H&E stained). Images show HMGB1, immunohistochemical expression, and localization. Images are representative of *n* = 3 skin sample. For H&E images: black horizontal scale bar = 100 μm (400× magnification). For HMGB1: black horizontal bar =60 μm (zoomed 40× whole slide scanned image). Dashed lines represent the dermal epidermal junction.

Having determined that TNF‐α induces HMGB1 release from a keratinocyte cell‐line, we looked at the effect of TNF‐α in a more physiologically relevant model, healthy human skin explants, to see whether in vitro observations could be reproduced. Exposure of healthy skin explants to TNF‐α for 72 h induced grade 2 epidermal toxicity compared to grade I toxicity in media‐only treated explants (Supporting Information Table S4 and Figure 3). Although administration of etanercept did not abate toxicity, TNF‐α induced loss of epidermal HMGB1 expression (which was not seen in media‐only controls) was negated by etanercept (Figure 3). Extracellular levels of HMGB1 in the supernatant (Supporting Information Figure S3) were significantly higher in TNF‐α treated explants (7.83 ng/mL ± 1.2) versus controls (1.03 ng/mL ± 0.14, *P* < 0.01). Etanercept co‐exposure with TNF‐α did not significantly alter HMGB1 levels (10.87 ng/mL ± 1.70, *P* > 0.05).

Anakinra (positive control)‐treated explants exhibited grade II/III toxicity or grade II in the presence of etanercept (Supporting Information Figure S3). HMGB1 supernatant levels were significantly higher in response to anakinra (59.64 ng/mL ± 20.17, *P* < 0.05) compared to untreated controls. Co‐administration of etanercept did not result in a significant change in HMGB1 concentrations in response to anakinra (12.13 ng/mL ± 5.54; ns).

RIPK3 expression in the basal layer was not significantly higher in TNF‐α‐treated explants compared to controls (Supporting Information Figure S1). However, there was a noticeable reduction in RIPK3 expression in explants (control and TNF‐α‐treated) co‐administered etanercept. No significant expression of cleaved caspase 3 was observed in any of the treatment groups (Supporting Information Figure S1).

### 3.3 HMGB1 expression and supernatant release in healthy skin explants exposed to SJS/TEN patient serum

Having established the effect of TNF‐α on epidermal toxicity and HMGB1 release, the ex vivo work was expanded to look at the effect of exposing healthy skin to sera from healthy control, lichenoid dermatitis and SJS/TEN patients (acute phase). TNF‐α concentrations of the three sera were determined by ELISA as healthy control 2.92 pg/mL (0.29 pg/mL final concentration), lichenoid dermatitis 9.17 pg/mL (0.92 pg/mL final concentration), and SJS/TEN 12.25 pg/mL (1.22 pg/mL final concentration). As previously described,^10^ exposure of explants to ICI‐induced SJS/TEN patient serum induced grade III pathology with intra‐epidermal damage and necrosis in both duplicates. The addition of etanercept reduced the pathology to grade II in both instances (Supporting Information Table S4). The addition of serum from a patient with ICI‐induced lichenoid dermatitis resulted in grade II pathology in both replicates with etanercept co‐administration having little effect. Exposure to serum from an ICI‐tolerant patient resulted in no significant difference in pathology (grade I) compared to media only control.

Epidermal HMGB1 expression in explants treated with SJS/TEN patient serum was significantly decreased (Figure 3) in both replicates, with levels being visibly higher when the explants were co‐incubated with etanercept. Conversely, apparent immune cell HMGB1 immunostaining in SJS/TEN serum‐treated explants was increased in the presence of etanercept (Figure 3). Explants treated with sera from either ICI‐tolerant or ICI‐induced lichenoid dermatitis patients showed no substantial loss of epidermal HMGB1 and no modulation of effect by etanercept. Cleaved caspase 3 expression was notionally elevated in SJS/TEN serum‐treated explants at the epidermal basal layer (Supporting Information Figure S2) compared to tolerant serum‐treated explants but this was not substantially modulated by etanercept. RIPK3 expression was significant in all explants in the basal layer and was visibly lower in TNF‐α and SJS/TEN serum‐treated explants compared to control, healthy, or lichenoid dermatitis serum treatment (Supporting Information Figures S1 and S2).

In addition to in situ skin HMGB1 expression, we also examined extracellular HMGB1 in the explant culture media (Supporting Information Figure S3). This was found to be significantly higher in explants treated with SJS/TEN patient serum (16.91 ng/mL ± 3.51) compared to the media‐only control (1.03 ng/mL ± 0.14, *P* < 0.05). There was no significant difference in those co‐administered etanercept (19.00 ng/mL ± 2.62; *P* < 0.05). Explants treated with sera from either the tolerant or lichenoid dermatitis patient (4.36 ng/mL ± 1.83 and 1.82 ng/mL ± 0.22, respectively) did not demonstrate higher mean supernatant HMGB1 levels compared to control explants (ns) and there was no significant difference from either when co‐administered etanercept (ns).

### 3.4 Quantitative digital image analysis of HMGB1 skin expression

**Figure 4:**
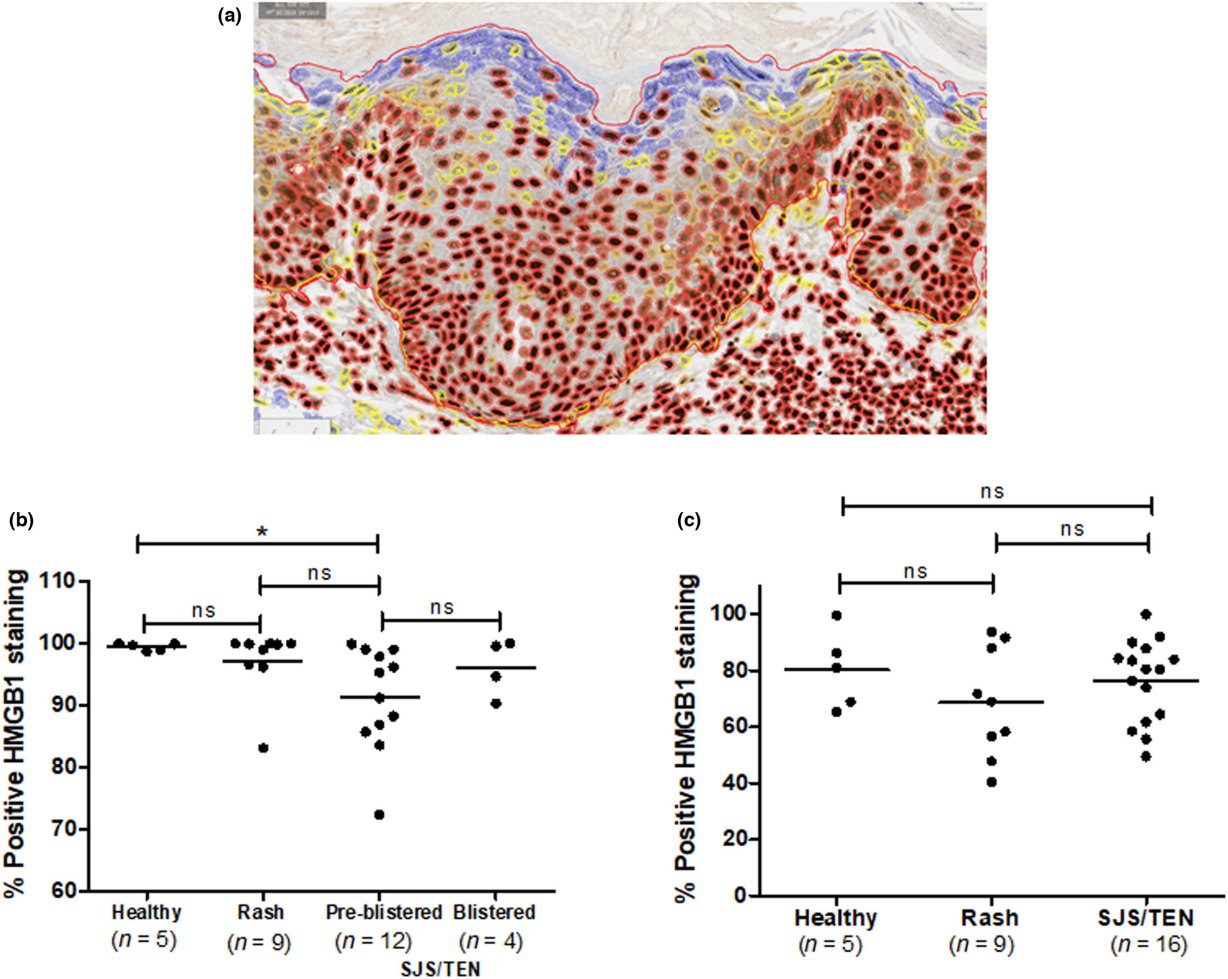
Digital whole slide image analysis of epidermal and dermal HMGB1 cell positivity in healthy, maculopapular exanthem, and SJS/TEN skin samples from the Cleveland cohort. (a) Exemplar cell detection‐visual deployment of the result. The border of the cells represents the following: blue, negative; yellow, weakly positive; orange, moderately positive; red, strongly positive (400× magnification). (b) Epidermal and (c) dermal cellular staining for HMGB1 cutaneous ADRs. Data represent percentage cells with positive HMGB1 staining (as visualized by immunohistochemistry) for healthy, rash, and SJS/TEN. Horizontal lines represent mean values. *P < 0.05, ns = not significant.

Previous work^1^, utilizing semiquantitative analysis of epidermal HMGB1 expression, showed a significant decrease in the SJS/TEN epidermis. We therefore used image analysis to quantify dermal and epidermal cellular HMGB1 expression in healthy, drug‐induced maculopapular exanthema, and SJS/TEN skin. Analysis of the skin biopsies demonstrated that HMGB1 positive cells in pre‐blistered epidermis from SJS/TEN patients (91.3% ± 2.4, mean ± SE) were significantly lower than in healthy controls (99.5% ± 0.3, *P* = 0.047; Figure 4b). HMGB1 positive cells were not significantly different in maculopapular exanthema skin (97.2% ±1.8) versus healthy control (*P* > 0.05). SJS/TEN samples with detached epidermis were analyzed separately by manual observation since the high levels of positive staining appeared to be an artifact of the analysis methodology. Dermal percentage HMGB1 positive cells (Figure 4c) were not significantly different between the healthy, maculopapular exanthema and SJS/TEN phenotypes.

## 4 DISCUSSION

The findings from this study show a clear link between TNF‐α‐induced cell death (predominantly necroptosis) and HMGB1 release in keratinocytes (Figures 1 and 2). Our previous observations^1^ showed that HMGB1 is released from keratinocytes in SJS/TEN even prior to epidermal detachment. The suggestion that RIPK3 necroptotic keratinocyte death may be a predominant source of HMGB1 (Figures 1 and 2) is consistent with previous literature suggesting that necroptosis is a key mediator of epidermal injury in severe skin‐blistering ADRs.^7^, ^8^ Our finding that TNF‐α plus BV‐6 elicits keratinocyte cell death (and HMGB1 release) both in vitro (Figure 1) and ex vivo (Figure 3) shows that TNF‐α, at least in part, mediates keratinocyte cell death in SJS/TEN, although this does not exclude the role of other complementary mechanisms, for example neutrophil‐derived LL‐37 cytotoxicity.^17^

Interestingly, loss of HMGB1 expression appears to be a more sensitive marker of epidermal injury than both RIPK3 and caspase expression in explants treated with either TNF‐α or SJS/TEN serum (Figure 3 and Supporting Information Figures S1 and S2). The data suggest that both TNF‐α and SJS/TEN serum lower RIPK3 expression and may be negatively correlated to HMGB1 expression in the epidermis. This is hypothesized to be due to the RIPK3 antibody used not cross‐reacting with the phosphorylated RIPK3 formed during necroptotic cell death.^18^ The lack of significant epidermal staining for cleaved caspase 3 is intriguing and seems to suggest that negligible apoptotic cell death is occurring. This supports the in vitro evidence that the predominant contributor to epidermal HMGB1 release is necroptotic cell death. We also observe apparent increased levels of HMGb1 expression in immune cells (Figure 3), which could also account for increased extracellular levels. This is supported by the observation that TNF‐α and SJS/TEN serum increased the number of HMGb1‐positive cell I the dermis and supports the suggestion that the processes driving increased HMGB1 are multifaceted. This would require validation by possibly determining co‐localization of HMGB1 with CD14+/CD16+ monocytes, which have been shown to play a key role in epidermal damage in SJS/TEN.^19^ Future experiments with a suitably powered number of explants should use digital pathology to undertake a quantitative correlation of this relationship. However, the data presented herein suggest that epidermal HMGB1 expression may represent a highly sensitive biomarker of SJS/TEN and cellular HMGB1 translocation, a robust end‐point marker for assessing keratinocyte cell death (predominantly necroptosis) in SJS/TEN pathogenesis.

The work presented in skin explant models demonstrates that TNF‐α can induce epidermal release of HMGB1 (Figure 3) and grade 2 toxicity (Supporting Information Table S4), which leads to elevated supernatant levels (Supporting Information Figure S3). However, whilst the addition of etanercept negated epidermal release, it did not reduce extracellular levels (Supporting Information Figure S3). TNF‐α is known to induce HMGB1 release from monocytes,^20^ and it is therefore possible that the extracellular levels of HMGB1 in SJS/TEN are due to TNF‐α‐induced release by both epidermal and immune cells, and are not only due to epidermal loss of expression. An alternative hypothesis is that etanercept itself increases HMGB1 release from activated immune cells^21^ which counters the inhibition of HMGB1 release from injured keratinocytes by etanercept. This also suggests that etanercept has an effect on both inflammatory cells and keratinocytes. This is also true of anakinra,^21^ which would explain the very large increase in HMGB1 that is seen and the lack of reduction in the presence of etanercept (Supporting Information Figure S3).

Epidermal expression of HMGB1 was significantly decreased by exposure to SJS/TEN patient serum at 1:10 dilution (Figure 3) and marginally restored by etanercept. This mirrored the effect on toxicity where a marginal decrease was seen in response to etanercept (Supporting Information Table S4 and Figure 3). This suggests that TNF‐α might play a role in mediating HMGB1 release and toxicity in SJS/TEN but HMGB1 release is also attributable to non‐TNF‐α‐mediated pathways. A limitation of our studies is the small number of patient samples tested so data should be assessed with caution. However, the results obtained were quite distinct between sera obtained from the SJS/TEN patient, and sera from the tolerant control and the patient with maculopapular exanthema. Further work will be needed to assess interindividual variability in response across multiple different explants and serum samples. It should be noted that the SJS/TEN case from which sera was used was an ICI‐treated patient. However, previous work has shown that the reaction was due to exposure to iodinated contrast media precipitated by ICI therapy rather than the actual ICI^9^ so the pathogenesis of this case is akin to a “normal” small‐molecule‐induced SJS/TEN phenotype.

The clinical benefit of TNF‐α inhibition by etanercept in treating SJS/TEN has been demonstrated in multiple case reports^3^ and in a randomized control trial.^4^ However, the actual mechanism of action of etanercept in SJS/TEN has not been established. We were unable to assess the role of etanercept on pathogenesis in the model in isolation or co‐administered with anakinra, which is a limitation although we would not expect any significant modulation in either scenario. Our findings suggest that etanercept, whilst attenuating TNF‐α‐induced keratinocyte death, also significantly reduces HMGB1 release (Figure 1). The suggestion that TNF‐α has a role in the SJS/TEN pathogenesis is not new^22^, ^23^ but the link to HMGB1 is novel and could represent a new insight into the downstream effects of TNF‐α and the mechanism of action of etanercept in SJS/TEN. It should be noted that the action of TNF‐α on keratinocytes in this model is not specific and indeed it is likely that, as part of the observed pathogenesis, it is mediating its effect via skin‐resident CD8+ T‐effector cells.^24^

We used digital pathology to build on previous observations of decreased epidermal HMGB1 expression in SJS/TEN skin.^1^ A statistically significant decrease in HMGB1 positive cells was observed in pre‐blistered SJS/TEN versus healthy skin (Figure 4), although the difference between maculopapular exanthema and SJS/TEN serum did not reach significance. The data confirm that HMGB1 nuclear > cytosol > extracellular translocation is indicative of early epidermal stress in SJS/TEN.

The data further underline the potential utility of epidermal HMGB1 release as an early marker of epidermal cell death, and potentially detachment as we have previously proposed.^1^ HMGB1 may also, through its isoform‐dependent immunomodulatory functions,^2^ exacerbate tissue damage. This will require further study. The data presented further suggest that HMGB1 keratinocyte release may be a viable proxy biomarker for the onset of SJS/TEN and that, at least in keratinocytes, it is related to both necroptotic and apoptotic cell death. Explant data suggest that epidermal HMGB1 may be a more sensitive marker than both RIPK3 and cleaved caspase, although there is a suggestive correlation between HMGB1 and RIPK3. Future work will look to further establish this is both treated explants and clinical biopsy samples.

We have shown that the exposure of healthy skin explants to both TNF‐α and serum from an SJS/TEN patient was able to evoke a skin phenotype akin to SJS/TEN in both morphology and HMGB1 expression. We have also shown that etanercept was able to attenuate toxicity and, at least for TNF‐α, reverse HMGB1 cellular release in the epidermis. The lack of effect of etanercept in SJS/TEN serum‐treated explants is likely to be due to the multitude of other molecules present which exert cytotoxic effects independently of TNF‐α, in keeping with the fact that the pathogenesis of SJS/TEN is multifaceted and complex. However, this physiologically‐relevant skin explant model has the potential to be used for assessing the pathogenic role and mechanism of other immune‐derived cytotoxic mediators implicated in SJS/TEN, such as granulysin, perforin, granzyme B, and LL37.^25^, ^26^, ^27^, ^28^ Furthermore, by inducing an SJS/TEN‐like event in healthy skin explants with TNF‐α, and inhibiting it with etanercept, we have a valid model system with the potential to be used for screening targeted therapies for potential therapy in SJS/TEN. The results presented here have be interpreted with some caution. Given the small sample size, it is not possible to determine interindividual variability in response, both between skin donors and between serum samples. This will be the focus of future work.

To conclude, we have demonstrated that keratinocyte‐derived HMGB1 release is a useful biomarker for keratinocyte injury in early SJS/TEN. The skin explant model described may be a useful model to identify not only other proteins involved not only in causing epidermal damage, but also in screening novel therapies to treat SJS/TEN. This will, however, need further validation.

## CONFLICT OF INTEREST STATEMENT

Etanercept (Benepali) was donated to DFC by Biogen Inc. The authors report no other conflicts of interest.

## Supporting information


Figure S1.
Figure S2.
Figure S3.
Table S1.
Table S2.
Table S3.
Table S4.

## References

[1] Carr DF , Wang CW , Bellon T , Ressel L , Nwikue G , Shrivastava V , et al. Serum and blister‐fluid elevation and decreased epidermal content of high‐mobility group box 1 protein in drug‐induced Stevens‐Johnson syndrome/toxic epidermal necrolysis. Br J Dermatol. 2019;181:166–74. 30613954 10.1111/bjd.17610 PMC6617791

[2] Andersson U , Antoine DJ , Tracey KJ . The functions of HMGB1 depend on molecular localization and post‐translational modifications. J Intern Med. 2014;276:420–4. 25346011 10.1111/joim.12309

[3] Zhang S , Tang S , Li S , Pan Y , Ding Y . Biologic TNF‐alpha inhibitors in the treatment of Stevens‐Johnson syndrome and toxic epidermal necrolysis: a systemic review. J Dermatolog Treat. 2020;31:66–73. 30702955 10.1080/09546634.2019.1577548

[4] Wang CW , Yang LY , Chen CB , Ho HC , Hung SI , Yang CH , et al. Randomized, controlled trial of TNF‐alpha antagonist in CTL‐mediated severe cutaneous adverse reactions. J Cutan Med Surg. 2018;128:985–96. 10.1172/JCI93349 PMC5824923 29400697

[5] Lee Y‐Y , Ko J‐H , Wei C‐H , Chung W‐H . Use of etanercept to treat toxic epidermal necrolysis in a human immunodeficiency virus‐positive patient. Dermatol Sin. 2013;31:78–81.

[6] Viard‐Leveugle I , Gaide O , Jankovic D , Feldmeyer L , Kerl K , Pickard C , et al. TNF‐alpha and IFN‐gamma are potential inducers of Fas‐mediated keratinocyte apoptosis through activation of inducible nitric oxide synthase in toxic epidermal necrolysis. J Invest Dermatol. 2013;133:489–98. 22992806 10.1038/jid.2012.330

[7] Saito N , Qiao H , Yanagi T , Shinkuma S , Nishimura K , Suto A , et al. An annexin A1‐FPR1 interaction contributes to necroptosis of keratinocytes in severe cutaneous adverse drug reactions. Sci Transl Med. 2014;6:245ra95. 10.1126/scitranslmed.3008227 25031270

[8] Panayotova‐Dimitrova D , Feoktistova M , Leverkus M . RIPping the skin apart: necroptosis signaling in toxic epidermal necrolysis. J Invest Dermatol. 2015;135:1940–3. 26174536 10.1038/jid.2015.159

[9] Hammond S , Olsson‐Brown A , Gardner J , Thomson P , Ali SE , Jolly C , et al. T cell mediated hypersensitivity to previously tolerated iodinated contrast media precipitated by introduction of atezolizumab. J Immunother Cancer. 2021;9:e002521. 34049931 10.1136/jitc-2021-002521 PMC8166637

[10] Olsson‐Brown A , Yip V , Ogiji ED , Jolly C , Ressel L , Sharma A , et al. TNF‐α mediated keratinocyte expression and release of matrix metalloproteinase 9: putative mechanism of pathogenesis in Stevens‐Johnson syndrome/ toxic epidermal necrolysis. J Invest Dermatol. 2023;143:1023–30.e.7. 36581093 10.1016/j.jid.2022.11.024

[11] Kaiser C , Knight A , Nordstrom D , Pettersson T , Fransson J , Florin‐Robertsson E , et al. Injection‐site reactions upon Kineret (anakinra) administration: experiences and explanations. Rheumatol Int. 2012;32:295–9. 21881988 10.1007/s00296-011-2096-3 PMC3264859

[12] Dudeck J , Kotrba J , Immler R , Hoffmann A , Voss M , Alexaki VI , et al. Directional mast cell degranulation of tumor necrosis factor into blood vessels primes neutrophil extravasation. Immunity. 2021;54:468–83.e5. 33484643 10.1016/j.immuni.2020.12.017

[13] Lerner KG , Kao GF , Storb R , Buckner CD , Clift RA , Thomas ED . Histopathology of graft‐vs.‐host reaction (GvHR) in human recipients of marrow from HL‐A‐matched sibling donors. Transplant Proc. 1974;6:367–71. 4155153

[14] Bankhead P , Loughrey MB , Fernández JA , Dombrowski Y , McArt DG , Dunne PD , et al. QuPath: open source software for digital pathology image analysis. Sci Rep. 2017;7:16878. 29203879 10.1038/s41598-017-17204-5 PMC5715110

[15] Feoktistova M , Geserick P , Kellert B , Dimitrova Diana P , Langlais C , Hupe M , et al. cIAPs block Ripoptosome formation, a RIP1/Caspase‐8 containing intracellular cell death complex differentially regulated by cFLIP isoforms. Mol Cell. 2011;43:449–63. 21737330 10.1016/j.molcel.2011.06.011 PMC3163271

[16] Peña‐Blanco A , García‐Sáez AJ . Bax, Bak and beyond ‐ mitochondrial performance in apoptosis. FEBS J. 2018;285:416–31. 28755482 10.1111/febs.14186

[17] Kinoshita M , Ogawa Y , Hama N , Ujiie I , Hasegawa A , Nakajima S , et al. Neutrophils initiate and exacerbate Stevens‐Johnson syndrome and toxic epidermal necrolysis. Sci Transl Med. 2021;13:eaax2398. 34193610 10.1126/scitranslmed.aax2398 PMC9392155

[18] Webster JD , Solon M , Haller S , Newton K . Detection of necroptosis by Phospho‐RIPK3 immunohistochemical labeling. Methods Mol Biol. 2018;1857:153–60. 30136239 10.1007/978-1-4939-8754-2_15

[19] Tohyama M , Watanabe H , Murakami S , Shirakata Y , Sayama K , Iijima M , et al. Possible involvement of CD14+ CD16+ monocyte lineage cells in the epidermal damage of Stevens‐Johnson syndrome and toxic epidermal necrolysis. Br J Dermatol. 2012;166:322–30. 21936856 10.1111/j.1365-2133.2011.10649.x

[20] Andersson U , Wang H , Palmblad K , Aveberger AC , Bloom O , Erlandsson‐Harris H , et al. High mobility group 1 protein (HMG‐1) stimulates proinflammatory cytokine synthesis in human monocytes. J Exp Med. 2000;192:565–70. 10952726 10.1084/jem.192.4.565 PMC2193240

[21] Schierbeck H , Wähämaa H , Andersson U , Harris HE . Immunomodulatory drugs regulate HMGB1 release from activated human monocytes. Mol Med. 2010;16:343–51. 20386869 10.2119/molmed.2010.00031 PMC2935946

[22] Caproni M , Torchia D , Schincaglia E , Volpi W , Frezzolini A , Schena D , et al. Expression of cytokines and chemokine receptors in the cutaneous lesions of erythema multiforme and Stevens‐Johnson syndrome/toxic epidermal necrolysis. Br J Dermatol. 2006;155:722–8. 16965421 10.1111/j.1365-2133.2006.07398.x

[23] Norman MU , Lister KJ , Yang YH , Issekutz A , Hickey MJ . TNF regulates leukocyte‐endothelial cell interactions and microvascular dysfunction during immune complex‐mediated inflammation. Br J Pharmacol. 2005;144:265–74. 15655512 10.1038/sj.bjp.0706081 PMC1576001

[24] Ye LL , Wei XS , Zhang M , Niu YR , Zhou Q . The significance of tumor necrosis factor receptor type II in CD8(+) regulatory T cells and CD8(+) effector T cells. Front Immunol. 2018;9:583. 29623079 10.3389/fimmu.2018.00583 PMC5874323

[25] Chung WH , Hung SI , Yang JY , Su SC , Huang SP , Wei CY , et al. Granulysin is a key mediator for disseminated keratinocyte death in Stevens‐Johnson syndrome and toxic epidermal necrolysis. Nat Med. 2008;14:1343–50. 19029983 10.1038/nm.1884

[26] Inachi S , Mizutani H , Shimizu M . Epidermal apoptotic cell death in erythema multiforme and Stevens‐Johnson syndrome. Contribution of perforin‐positive cell infiltration. Arch Dermatol. 1997;133:845–9. 9236522

[27] Nassif A , Bensussan A , Dorothée G , Mami‐Chouaib F , Bachot N , Bagot M , et al. Drug specific cytotoxic T‐cells in the skin lesions of a patient with toxic epidermal necrolysis. J Invest Dermatol. 2002;118:728–33. 11918724 10.1046/j.1523-1747.2002.01622.x

[28] Kimura H , Hasegawa A , Takei I , Kawai T , Tsuchida Y , Abe Y , et al. Characteristic pathological features of keratinocyte death in a case of Stevens‐Johnson syndrome manifested by an immune checkpoint inhibitor. J Eur Acad Dermatol Venereol. 2021;35:e142–5. 32780890 10.1111/jdv.16872

