# Supplementary material for: TNF‐α induced extracellular release of keratinocyte high‐mobility group box 1 in Stevens‐Johnson syndrome/toxic epidermal necrolysis: Biomarker and putative mechanism of pathogenesis
Source: J Dermatol. 2023 Jun 2;50(9):1129–39. doi: 10.1111/1346-8138.16847 (PMC10947163; doi:10.1111/1346-8138.16847)
Supplement: Supplementary file 1 — Figure S1. Figure S2. Figure S3. Table S1. Table S2. Table S3. Table S4. [file JDE-50-1129-s001.docx]

**Supplementary INFORMATION**

**Materials and Methods**

**Table S1**. Oligo nucleotide primer sequences for PCR amplification of cDNA sequences containing Sfil enzyme restriction sites (in red).

| Bak | Forward | 5’ AAAGGCCTCTGAGGCCACCATGGCTTGGAGCCAC-3’ |
| --- | --- | --- |
|  | Reverse | 5’ GCTTGGCCTGACAGGCCTCATGATTTGAAGAATCTTCGTACCAC 3’ |
| RIPK3 | Forward | 5’ AAAGGCCTCTGAGGCCACCATGTCGTGCGTCAAGTTATG 3’ |
|  | Reverse | 5’ AAGCTTGGCCTGACAGGCCTCATGCTTTTTCGAACTGGGGGTGGC 3’ |
| MLKL | Forward | 5’ AAGGCCTCTGAGGCCACCATGGAAAATTTGAAGCATATTATCACC 3’ |
|  | Reverse | 5’ AAGCTTGGCCTGACAGGCCTCATGCCTTTTCGAACTGCGGGTGGC 3’ |

**Table S2**. Clinical characteristics of ICI-induced cutaneous ADR patient case series.

| **Centre** | **ID** | **Age** | **Gender** | **SAPhenotype** | **Causal Drug** | **IHC HMGB1** | **Serum exposed to explants** |
| --- | --- | --- | --- | --- | --- | --- | --- |
| Liverpool | HIE_COH_001 | 44 | M | SJS/TEN | atezolizumab |  | Y |
| Liverpool | HIE_COH_007 | 71 | F | Lichenoid Dermatitis | pembrolizumab |  | Y |
| Liverpool | C37 V5 | 70 | F | Drug Tolerant Control | ipilimumab/ nivolumab |  | Y |
| Cleveland | MMST-001 | 68 | M | Normal | n/a | Y |  |
| Cleveland | MMST-002 | 24 | F | TEN | ketorolac | Y |  |
| Cleveland | MMST-004 | 62 | M | MPE | piperacillin / tazobactam | Y |  |
| Cleveland | MMST-005 | 39 | F | SJS/TEN | phenytoin | Y |  |
| Cleveland | MMST-006 | 54 | M | SJS/TEN | vancomycin | Y |  |
| Cleveland | MMST-008 | 23 | F | SJS | trimethoprim / sulfamethoxazole | Y |  |
| Cleveland | MMST-009 | 51 | F | SJS | vancomycin | Y |  |
| Cleveland | MMST-010 | 62 | M | SJS | piperacillin / tazobactam | Y |  |
| Cleveland | MMST-011 | 28 | F | TEN | trimethoprim / sulfamethoxazole | Y |  |
| Cleveland | MMST-012 | 22 | M | MPE | undetermined | Y |  |
| Cleveland | MMST-013 | 78 | F | MPE | undetermine | Y |  |
| Cleveland | MMST-014 | 54 | M | SJS/TEN | amiodarone | Y |  |
| Cleveland | MMST-015 | 58 | F | SJS/TEN | vancomycin | Y |  |
| Cleveland | MMST-016 | 48 | M | SJS/TEN | trimethoprim / sulfamethoxazole | Y |  |
| Cleveland | MMST-018 | 30 | M | SJS/TEN | voriconazole | Y |  |
| Cleveland | MMST-019 | 71 | F | SJS/TEN | valaciclovir | Y |  |
| Cleveland | MMST-020 | 55 | M | MPE | meloxicam | Y |  |
| Cleveland | MMST-021 | 84 | M | MPE | piperacillin / tazobactam | Y |  |
| Cleveland | MMST-022 | 90 | M | Normal | n/a | Y |  |
| Cleveland | MMST-023 | 29 | F | SJS/TEN | co-amoxiclav | Y |  |
| Cleveland | MMST-024 | 10 | M | SJS/TEN | ibuprofen | Y |  |
| Cleveland | MMST-025 | 57 | M | SJS/TEN | trimethoprim / sulfamethoxazole | Y |  |
| Cleveland | MMST-026 | 79 | M | SJS/TEN | valcyclovir | Y |  |
| Cleveland | S12-6309 | 61 | M | Drug Reaction | piperacillin /tazobactam | Y |  |
| Cleveland | S13-103528 | 59 | F | Drug Reaction | phenytoin | Y |  |
| Cleveland | S14-92010 | 59 | F | Drug Reaction | aspirin/lisionpril | Y |  |
| Cleveland | S12-24852 | 23 | F | Normal | n/a | Y |  |
| Cleveland | S14-82476 | 81 | F | MPE | phenytoin | Y |  |
| Cleveland | S12-24928 | 61 | f | Normal | n/a | Y |  |

**Table S3***.* Antibodies and dilutions used in western blotting for the detection of target proteins

| **Antibody (Product number)** | **Supplier** | **Species** | **Dilution** |
| --- | --- | --- | --- |
| MLKL (14993) | Cell Signalling Technologies | Rabbit | 1:1,000 |
| Caspase 3 (9661) | Cell Signalling Technologies | Rabbit | 1:1,000 |
| PARP (9542) | Cell Signalling Technologies | Rabbit | 1:1,000 |
| HMGB1 (ab18256) | Abcam | Rabbit | 1:5,000 |
| B-actin (AC-15) (Ab6276) | Abcam | Mouse | 1:20,0000 |
| StrepMab -Classic (2-1507-001) | IBA | Mouse | 1:4,000 |
| Anti-mouse IgG HRP conjugate (7076S) | Cell Signalling Technologies | Horse | 1:10,000 |
| Anti-rabbit IgG HRP conjugate (A0545) | Sigma-Aldrich | Goat | 1:10,000 |

**RESULTS**

**Table s4.** Histological scoring of treated healthy explants according to the Lerner scoring criteria (I-IV). A and B represent 2 individual treated skin explants.

|  | **No etanercept** | | | **+ 1µg/ml etanercept** | | | |
| --- | --- | --- | --- | --- | --- | --- | --- |
|  | **Lerner Score** | | **Observations** |  | **Lerner Score** | | **Observations** |
|  | **A** | **B** |  |  | **A** | **B** |  |
| **AB Media only** | I | I | - |  | - | - |  |
| **TNF-α (1ng/ml)** | II | II | - |  | II | II | - |
| **Anakinra (10µg/ml)** | II | III | Intra-epidermal/cleft formation/  damage + necrosis |  | II | II | Necrosis |
| **Serum-Free Media** | I | I | - |  | - | - | - |
| **Tolerant Serum (1:10)** | I | I | - |  | I | I | - |
| **Lich Derm Serum (1:10)** | II | II | - |  | II | I | - |
| **SJS/TEN Serum (1:10)** | III | III | Intra-epidermal/cleft formation/  damage + necrosis |  | II | II | Necrosis |

**
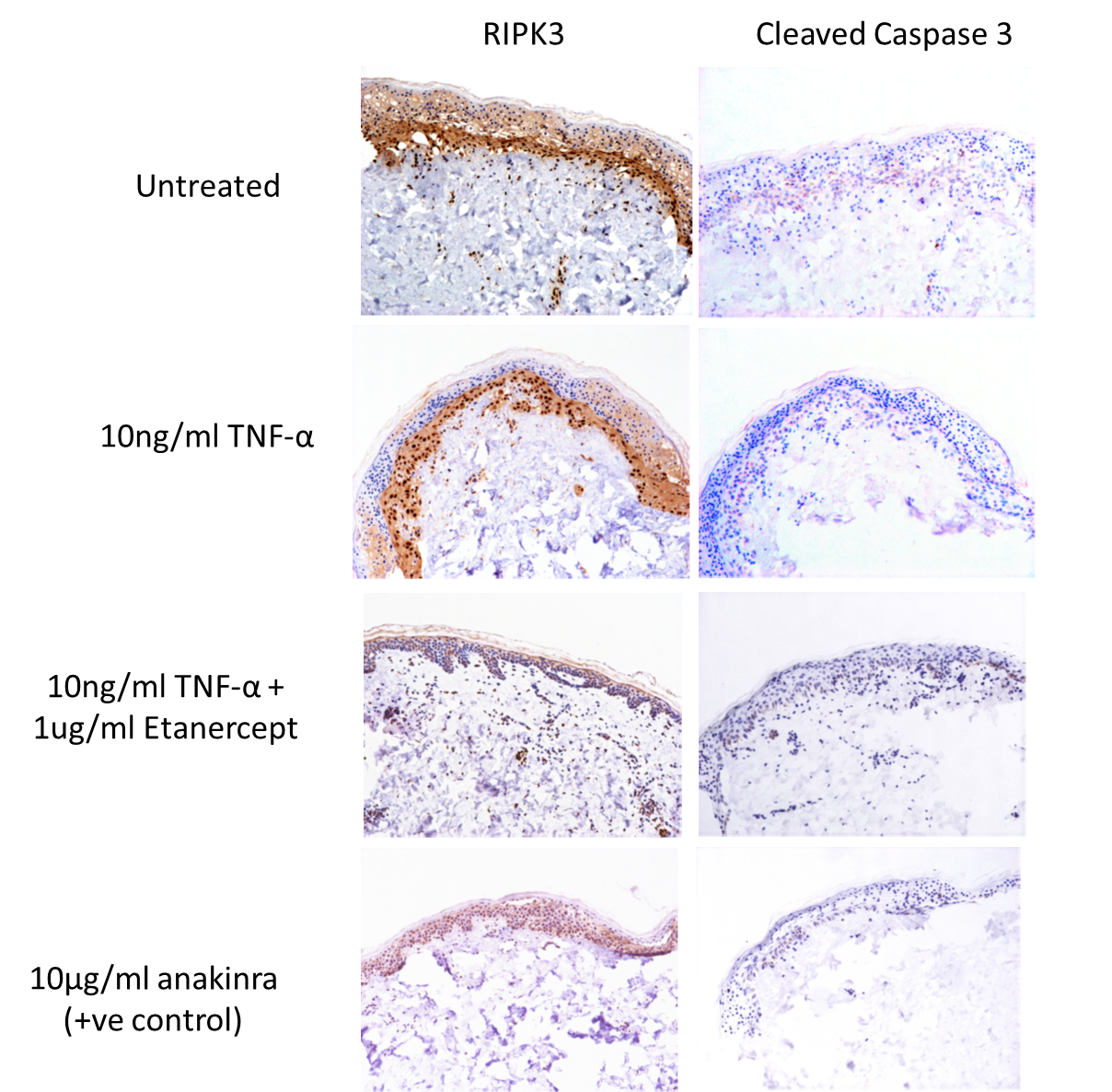
**

**Figure s1.** Effect of exposure TNF-α (acute reaction phase) on healthy skin explant RIPK3 and caspase 3 immunohistochemical expression and localisation +/- etanercept. Epiderml detachment positive control (anakinra) are also shown. Images are representative of n=3. 400x magnification.

**
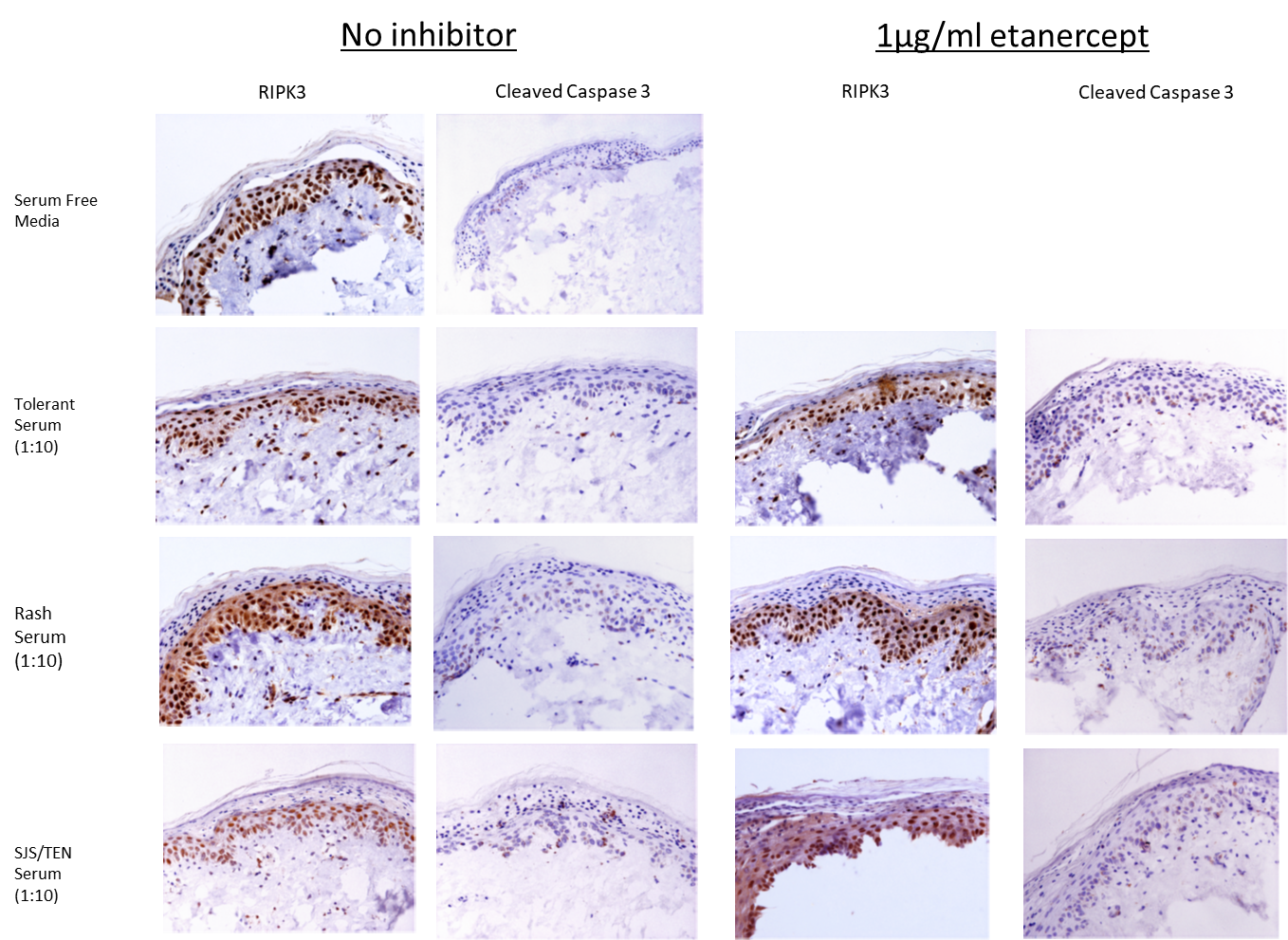
**

**Figure s2**. Effect of exposure to cutaneous ADR –patient serum (acute reaction phase) on healthy skin explant RIPK3 and caspase 3 immunohistochemical expression and localisation +/- etanercept. Images are representative of n=3. 400x magnification.

**Figure s3.** HMGB1 concentration in culture supernatant from healthy skin explants treated for 72 hours with ADR patient serum (control, lichenoid dermatitis or SJS/TEN) or TNF-α +/- etanercept (n=3, *=p<0.05, **=p<0.01 compared to untreated control, ns= no significant difference between + and - etanercept).
